# Combination of Synonymous and Missense Mutations in *JAK3* Gene Contributes to Severe Combined Immunodeficiency in One Child

**DOI:** 10.1155/2023/6633251

**Published:** 2023-09-13

**Authors:** Xingcui Wang, Rujin Tian, Haozheng Zhang, Mohnad Abdalla, Lu Bai, Yuqiang Lv, Min Gao, Guiyu Lin, Qinghua Liu, Yi Liu, Qiuxia He, Dong Wang, Kaihui Zhang

**Affiliations:** ^1^Science and Technology Service Platform, Qilu University of Technology (Shandong Academy of Sciences), China; ^2^Department of Rheumatology and Immunology, Children's Hospital Affiliated to Shandong University, Jinan, China; ^3^Pediatric Research Institute, Children's Hospital Affiliated to Shandong University, Jinan, China; ^4^Department of Pediatrics, The Second Hospital of Shandong University, Jinan, China; ^5^Department of Ultrasonic Imaging, Children's Hospital Affiliated to Shandong University, Jinan, China

## Abstract

Janus kinase 3 (JAK3, NP_000206.2) is a member of the Janus kinase (JAK) family of tyrosine kinases involved in cytokine receptor-mediated intracellular signal transduction JAK/STAT pathway. *JAK3* gene variants can lead to autosomal recessive severe combined immunodeficiency (SCID), which is T-cell-negative, B-cell-positive, and NK-cell-negative (OMIM: 600802). We have detected one infant suffering from cytomegalovirus, fever, and impaired respiratory function with low lymphocytes and immunoglobulin. Two compound heterozygous variants, c.1914G>T (p.L638=) and c.1048C>T (p.R350W), were identified in the proband, each of which was inherited from one unaffected parent. Analysis of splicing was carried out by the Sanger sequencing and RT-PCR from peripheral blood and a minigene splicing assay which both showed a deletion of exon 14 (128 bp) resulting from the c.1914G>T variant at the mRNA level. Bioinformatic analysis for the reported c.1048C>T (p.R350W) variant suggests that the variant is pathogenic. Based on the clinical characteristics of the patient and the functional verification of the gene variants, our pediatricians finally have diagnosed the infant as SCID (OMIM: 600802). The study is the first study regarding a synonymous variant of *JAK3* gene influencing alternative splicing. Our findings expand the mutation spectrum leading to JAK3 deficiency-related diseases and provide exact information for genetic counseling.

## 1. Introduction

Janus kinase 3 (JAK3, NP_000206.2) is a member of the Janus kinase (JAK) family of tyrosine kinases involved in cytokine receptor-mediated intracellular signal transduction. In this pathway, Janus kinase/signal transducers and activators of transcription (JAK/STAT) drive the development, differentiation, and function of immune cells and play a crucial role in blood cell formation [1, 2]. There are four JAK family members—JAK1, JAK2 (OMIM: 147796), JAK3 (OMIM: 600173), and tyrosine kinase-2 (TYK2; OMIM: 176941)—and 7 STAT proteins. All the JAK family members exhibit broad patterns of expression, except JAK3, in which expression is exclusively restricted to leukocytes. Leukocyte-specific JAK3 exclusively is associated with the IL-2 receptor *γ*-chain (*γ*_C_), which also serves as a component for the receptors of several lymphotropic cytokines, including IL-4, IL-7, IL-9, IL-15, and IL-21. In particular, IL-7 signaling is indispensable for T-cell development in humans, whereas IL-2 promotes antigen-driven specific T-cell expansion and peripheral T-cell homeostasis. IL-15 is essential for differentiation of NK cells. Moreover, IL-4 is vital for terminal B-cell differentiation and isotype switching. Therefore, due to the critical roles that *γ*_C_ and JAK3 play in the lymphoid activity, the patients with JAK3 deficiency typically lack both T and NK lymphocytes but have an almost normal number of B lymphocytes that nevertheless with functional deficiency. Therefore, the functional abnormality of JAK3 could give rise to T-cell-negative, B-cell-positive, and NK-cell-negative autosomal recessive severe combined immunodeficiency (SCID) (OMIM: 600802) (5) [3].

**Figure 1:**
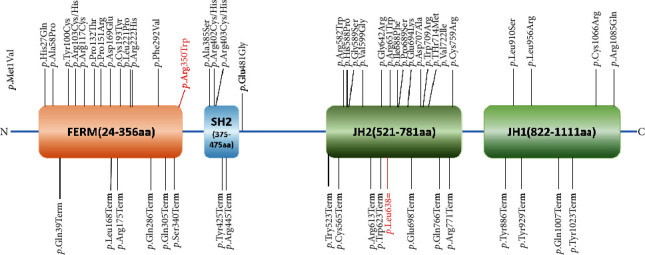
Schematic diagram for JAK3 and landscape of point mutations. JAK3 contains 4 important domains, FERM, SH2, JH2, and JH1. There are 7 JAK homology (JH) domains scattered across the protein that form the 4 critical domains. The reported 57 point mutations in JAK3 are shown over the diagram. The variants reported are shown in black, and the variant identified in this study is in red. The four JAK3 domains are FERM (four-point-one, ezrin, radixin, moesin homology domain, 24-356 bp), SH2 (src-homology 2, 375-475 bp), JH2 (Janus homology2, 521-781 bp, pseudokinase domain), and JH1 (822-1111 bp, tyrosine kinase domain).

The *JAK3* gene (NM_000215.3) is located on chromosome 19p12-13.1 with an ORF of 3372 bp. The JAK3 protein is an intracellular kinase of about 125 kDa and is predominantly expressed in cells of the hematopoietic system and binds to the *γ*_C_ [4]. By Jan 2023, there were 86 *JAK3* mutations reported related to immunodeficiency in the HGMD database (https://www.hgmd.cf.ac.uk/ac/index.php) (Figure 1). In this study, we report an infant suffering from cytomegalovirus infection, fever, and impaired respiratory function with low lymphocyte and immunoglobulin, diagnosed as SCID with two compound heterozygous mutations in the *JAK3* gene, c.1914G>T (p.L638=) and c.1048C>T (p.R350W). We confirmed the pathogenicity of the synonymous mutation c.1914G>T (p.L638=), demonstrating that this mutation results in abnormal splicing. This is the first time to report that a synonymous mutation of *JAK3* gene could affect the alternative splicing.

### 1.1. Patient

The patient, female, was admitted to the hospital on March 9, 2021, with a cough for 1 week and intermittent fever for 5 days. Physical examination: the respiratory sounds in both lungs are rough with slight moist rales audible. Chest X-ray showed soared, disordered, and blurry markings in both lungs and multiple patchy and blurry shadows in both lung fields, suspected of diagnosis of pneumonia. Cefotiam and erythromycin (2 days), ambroxol and vitamin K1 (1 day), dexamethasone (2 days), and budesonide and terbutaline (3 days) were given for 5 days. On the fifth day of the treatment, the infant's body temperature was normal, and after 1 day, she still had fever, and her cough was more severe than before, showing increased frequency, and she had wheezing 3 days ago, without dyspnea and cyanosis. Later, virus DNA detection was applied and indicated cytomegalovirus (CMV) infection. The child's dyspnea progressively worsened, manifesting as shallow shortness, suffocation, moaning, spitting, and bruising of the lips and limbs, but the body temperature was normal. Therefore, the infant was transferred to ICU. Respiratory pathogen nucleic acid testing ruled out popular respiratory bacterial infections and phlegm culture, and identification showed normal flora in the respiratory tract, and fecal culture and identification did not detect Salmonella and Shigella. Urinary routine examination showed positive urine protein; fecal routine examination showed a few white blood cells in the stool, a few fat globules, and a positive occult blood test. Electrocardiogram presented sinus rhythm and sinus tachycardia, and cardiac ultrasound showed patent foramen ovale. T lymphocyte was significantly lower than normal, and B lymphocyte was slightly lower. However, the function of B lymphocyte was damaged, and the immunoglobulin test showed that both immunoglobulin G (IgG) and IgA were both lower than normal. Taken together, the immunological results suggest that the child has low cellular and humoral immune functions. Therefore, immunoglobulin assistance therapy has been given. The cell morphology illustrates that some granulocytes have big granules, and there are middle late granulocytes. Heterotypic lymphocytes (reactive lymphocytes) account for 4%. There are nucleated red blood cells (5/100). Enlargement of mesenteric lymph nodes (multiple hypoechoic nodules detected, with the largest being approximately 1.2 × 0.7 cm) was detected, and no obvious enlarged lymph nodes have been detected on the neck by ultrasound of the intestinal and neck. Finally, the patient was diagnosed as pneumonia, respiratory dysfunction, cytomegalovirus infection, and immune deficiency diseases.

The patient's condition worsened, and the respiratory function was deteriorating, with chest X-rays showing increasingly severe pulmonary infections. The final clinical diagnosis is respiratory failure, severe pneumonia, cardiac insufficiency, and cytomegalovirus infection. Her condition was considered critical, and a ventilator was provided to assist breathing. The following medications were given to improve her condition, including albumin, furosemide to diuresis, midazolam to sedation, milrinone to improve circulation, meropenem to anti-infection, ganciclovir to antivirus, and methylprednisolone to anti-inflammatory and reduce inflammatory reactions: ambroxol for resolving phlegm and promoting sputum excretion; nebulized beclomethasone propionate, ipratropium bromide, and salbutamol for local anti-inflammatory and bronchodilation; glycyrrhetinic acid monoammonium cysteine to protect the liver; phosphocreatine to nourish the myocardium; and immunoglobulin to increase immunity. However, the patient's condition continues to worsen, with irreversible acidosis and heart failure, ultimately leading to death at 31 days after admission.

The infant was highly suspected to suffer the immunodeficiency disease based on the clinical features of sustained fever, pneumonia, cytomegalovirus infection, and low cellular and humoral immune functions. Infant immunodeficiency is mostly triggered by monogenic genetic diseases. Therefore, screening for whole exome sequencing was applied for pathogenesis. The infant was the first baby for the family, and full term to caesarean birth. The parents are not close relatives and are both healthy, and there are no similarly affected patients in the family.

## 2. Material and Methods

### 2.1. Compliance with Ethical Standards

This work was approved by the Medical Ethics Committee of the Children's Hospital affiliated to Shandong University (Ethics Approval No. ETYY-2014012). Clinical and laboratory examinations were performed on the proband after the written informed consent was obtained from her parents or guardians. All procedures in this study were performed in accordance with the Helsinki Declaration.

### 2.2. Next Generation Sequencing (NGS) and Variant Calling

Blood samples of the proband and her parents were obtained for DNA extraction using the QIAamp DNA Blood Midi Kit (Qiagen, Shanghai, China) according to the manufacturer's protocol. The extracted DNA concentration was determined using a NanoDrop 2000 ultraviolet spectrophotometer (Thermo Fisher, USA). Using the NovaSeq 6000 platform (Illumina, United States), whole exome sequencing (WES) with the Human Exome Probes P039-Exome (MyGenostics, Beijing, China) was used to screen for variants in the proband. The obtained mean exome coverage of the target regions was greater than 95% (>10 coverage; mean depth of over 100x). The Burrows-Wheeler Aligner software (BWA version 0.7.10) was used to align the sequencing data with human reference genome (hg19). SAM files were subsequently converted to BAM. GATK's RealignerTargetcreator and IndelRealigner were applied to local realignment, GATK's BaseRecalibratorbase was applied to quality score recalibration, and variants were jointly called using GATK's HaplotypeCaller in the “GENOTYPE_GIVEN_ALLELES” mode. After that, SNPs and indels were filtered using GATK's VariantFiltration, and ANNOVAR was used to annotate the variants. To remove popular variants (suballelic frequency > 5%), variant frequencies were determined in 1000 genomes, ExAC, gnomAD, ESP6500, and in-house databases. Potentially disease-causing variants associated with the patient-standardized HPO phenotype were prioritized for this study. The pathogenicity of novel variants was evaluated using SIFT, PolyPhen-2, MutationTaster, SpliceAI, and REVEL. Moreover, the variations reported in HGMD (http://www.hgmd.cf.ac.uk) and ClinVar (http://www.ncbi.nlm.nih.gov/clinvar) will be anatomized further. The variants identified in this study were classified according to the 2015 American College of Medical Genetics and Genomics (ACMG) guidelines [5].

### 2.3. Sanger's Sequencing to Verify the Variants

The likely pathogenic variants identified by WES in the proband using specific primers were validated by the Sanger sequencing. The reference sequence NM_000215 of *JAK3* was used. The Sanger validation primer sets were designed using Primer Premier v5.0 software. PCR amplification was performed using the AmpliTaq Gold 360 DNA polymerase (Applied Biosystems). The PCR products were further purified and sequenced using an ABI Prism 3700 automated sequencer (Applied Biosystems, Foster City, CA, USA).

### 2.4. Bioinformatic Analysis

Conservation analyses among multiple diverse species were performed using ClustalX software. Modeling of wild-type and mutant proteins was performed using the online SWISS-MODEL tool (http://www.swisshttp://model.expasy.org/). Splicing patterns of putative splicing variants were predicted using online RNA Splicer tool (https://rddc.tsinghua-gd.org/search-middle?to=SplitToolModel) and SpliceAI.

### 2.5. RNA Extraction, cDNA Obtained, and Sanger's Sequencing

Total RNA extracted from the peripheral blood of the proband was stored in TRIzol reagent (Invitrogen, USA) according to the manufacturer's protocol. First, peripheral blood cells were lysed by mixing in TRIzol reagent, a monophasic solution of phenol, guanidine isothiocyanate, and other proprietary components. TRIzol reagent maintains the integrity of the RNA because of the highly effective inhibition of RNase activity while disrupting cells and dissolving cell components during sample homogenization. Next, chloroform was added to separate the solution into aqueous and organic phases. RNA remained in the aqueous phase and was recovered by precipitation with isopropanol. The resulting nucleic acids were quantified using a NanoDrop 2000 ultraviolet spectrophotometer (Thermo Fisher Scientific, USA). The cDNA was obtained from RNA reverse transcription using the Takara PrimeScript™ RT Reagent Kit (Takara) following the manufacturer's instructions. The sequenced fragments, including the variant c.1914G>T (p. L638=) of the *JAK3* gene, were amplified from the cDNA of the proband using the forward primer *JAK3*-F (5′-TCATTCCTGGAAGCAGCGAGCTTG-3′) and a reverse primer *JAK3*-R (5′-CTTAGCAGGATCCAGGGCACTGATG-3′). Later, the possible cDNA alterations caused by the c.3453C>T variant were detected by the Sanger sequencing, as described above.

### 2.6. Minigene Splicing Assay

**Figure 2:**
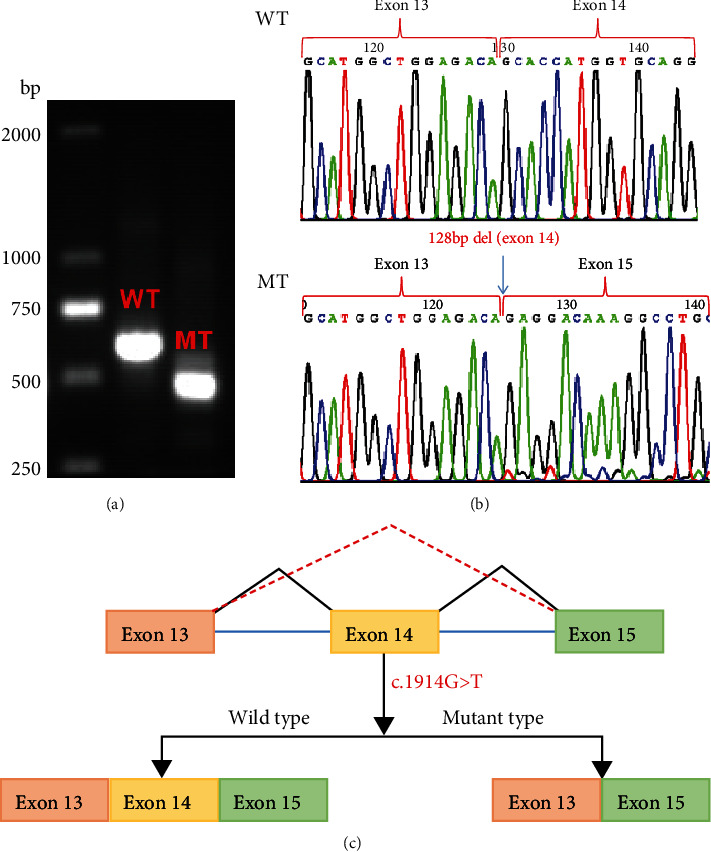
Splicing study of JAK3 c.1914G>T by minigene assay. (a) Agarose gel electrophoresis results of RT-PCR for the plasmid expression: the expected wild-type fragment is 546 bp, and the mutation-type fragment is 418 bp. (b) The Sanger sequencing of RT-PCR for the plasmid expression. (c) Schematic of splicing for JAK3 c.1914G>T. WT: wild type; MT: mutation type.

To verify the probable splicing effects caused by the c.1914G>T variant, a minigene splicing assay was performed in vitro. The minigene regions of the *JAK3* gene spanning exon 13, intron 13, exon 14, intron 14, exon 15, intron 15, and exon 16 (Figure 2) were amplified from the gDNA of the control using a forward primer (5′-AAGCTTGGTACCGAGCTCGGATCCTCATTCCTGGAAGCAGCGAGCTTG-3′) with the restriction site BamHI and a reverse primer (5′-TTAAACGGGCCCTCTAGACTCGAGCTTAGCAGGATCCAGGGCACTGATG-3′) with the restriction site XhoI. The amplified products were cloned into the pcDNA3.1 vector using a ClonExpress II One Step Cloning Kit (Vazyme, Nanjing, China). The cloned wild-type plasmid was validated using the Sanger sequencing. Mutant plasmids were created by recombining the mutant fragments obtained with the mutagenesis primers *JAK3*-MT-F (5′-CTATCTtGTGAGTGCTCCTCTGCCTGCTCCAC-3′) and *JAK3*-MT-R (5′-GAGCACTCACaAGATAGTTGAGGGCGTAGGCC-3′). The mutant plasmid was validated using the Sanger sequencing. The selected recombinant plasmids were transiently transfected into HEK293T cells using Lipofectamine 3000 (Invitrogen) following the manufacturer's instructions. Total RNA was extracted from cells cultured for 48 h using TRIzol reagent (Invitrogen, USA). RT-PCR was conducted using the MiniRT-F primer pair (5′-GGCTAACTAGAGAACCCACTGCTTA-3′) and MiniRT-R (5′-GTGGGAGGAGAGGTGAGTACTGTA-3′). We analyzed the amplified PCR fragments by agarose gel electrophoresis and identified the isoforms by the Sanger sequencing.

## 3. Results

### 3.1. Genetic Analysis

**Figure 3:**
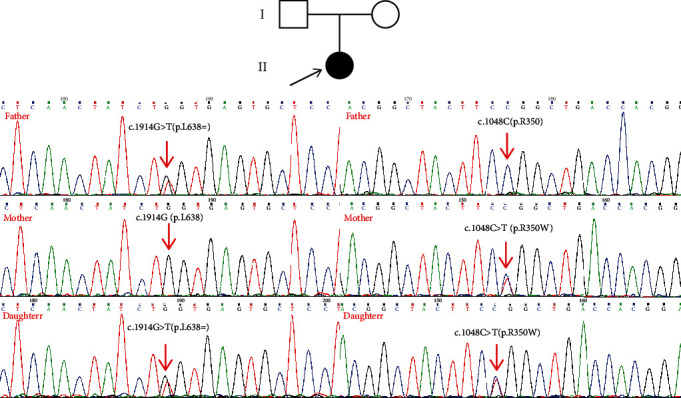
The Sanger sequencing results of the variant c.1048C>T (p.R350W). The Sanger sequencing showed that c.1048C>T (p.R350W) was maternally inherited.

According to clinical phenotype of immunodeficiency disorders and the disease genetic models, compound heterozygous variants (c.1914G>T and c.1048C>T, NM_000215) detected by clinical exome sequencing were suspected to be disease-associated variants in the proband in the *JAK3* gene, a known gene that can result in the autosomal recessive disease *severe combined immunodeficiency*, *T-cell-negative*, *B-cell-positive*, and *NK-cell-negative* (OMIM: 600173). The missense variant c.1048C>T (p.R350W) was reported in published literature [6]. *JAK3* c.1914G>T (p.L638=), a synonymous mutation, was absent from the 1000 Genomes Project, ExAC, ESC6500, and in-house database. Parental Sanger sequencing (Figure 3) illustrated that c.1914G>T (p.L638=) was a paternal variant, while c.1048C>T (p.R350W) was a maternal variant.

### 3.2. Bioinformatic Analysis

**Figure 4:**
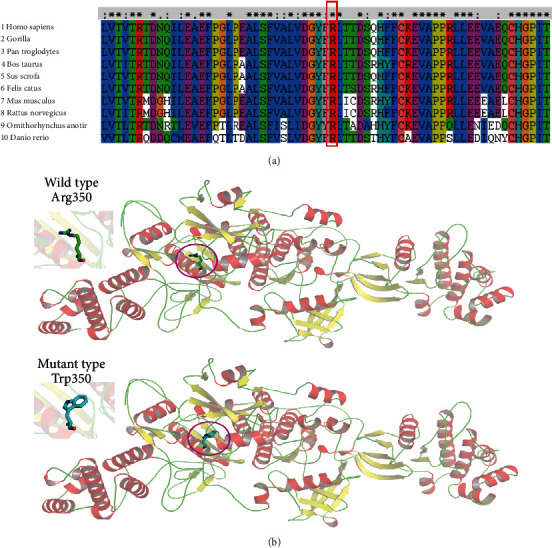
Pathogenicity of the missense mutations c.1048C>T (p.R350W) and the synonymous mutation c.1914G>T (p.L638=) of JAK3. (a) In silico analysis of p.Arg350Trp in JAK3 shows that the site p.Arg350 is highly conserved across species. (b) 3D structure of wild type and mutant type of p.Arg350Trp in JAK3, and the variant of p.Arg350Trp changes the side strand structure.

Although the missense variant c.1048C>T (p.R350W) was previously reported, its pathogenicity has not been further studied. Therefore, we analyzed the pathogenicity of the variant as predicted by bioinformatics. Conservation analysis revealed that the arginine at site p.R350 was highly conserved in multiple species (Figure 4(a)). Structural modeling showed changes in the side strand structure at residue 350, in which arginine was substituted by tryptophan (Figure 4(b)). The c.1048C>T (p.R350W) variant of *JAK3* was predicted to be deleterious by MutationTaster (0.999, disease-causing), REVEL, SIFT (0.005, damaging), and PolyPhen-2 (1.000, probably damaging). Since the synonymous variant c.1914G>T (p.L638=) was located near the exon-intron junction, an online RNA splicer tool (SpliceAI) was applied to assess the potential impact of the mutation on the splicing of this variant. The delta score of the c.1914G>T variant (which is interpreted as the probability of the variant being splice-altering) was 0 indicating that the tool did not predict altered splicing (Supplementary Figure 2).

### 3.3. Splicing Study of *JAK3* c.1914G>T by RT-PCR and Sanger's Sequencing from Proband Blood

**Figure 5:**
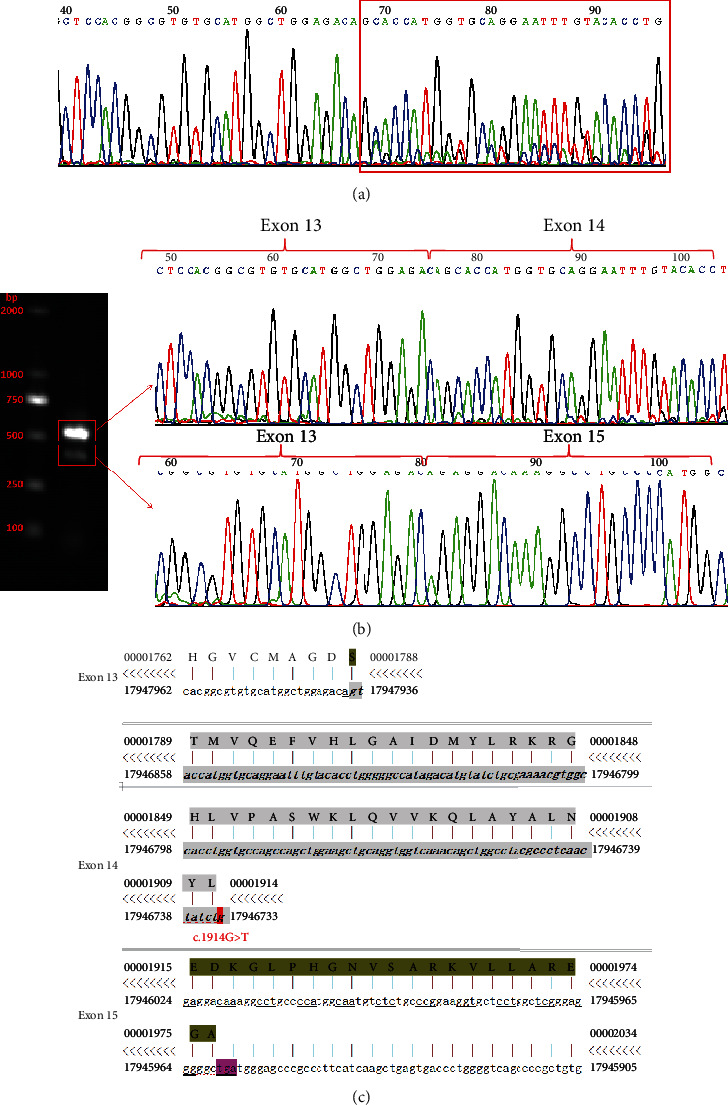
Splicing study of JAK3 c.1914G>T (p.L638=). (a) The Sanger sequencing of RT-PCR products based on peripheral blood samples of the proband. (b) The Sanger sequencing of the purified PCR products after electrophoresis on agarose gels. (c) Schematic of splicing sequence for JAK3 c.1914G>T; gray nucleotides were the deletion of exon 14 (128 bp), and new triplet codons were marked by subscript black and dashed red lines.

We nevertheless proceeded to investigate the consequence of the variant on splicing. First, we discovered that *JAK3* mRNA was highly expressed in peripheral blood mononuclear cells (PBMCs) using the UCSC database (http://genome.ucsc.edu/) and the Human Protein Atlas (https://www.proteinatlas.org/), an online tool (Supplementary Figure 1). The splicing consequence caused by *JAK3* c.1914G>T (p.L638=) was confirmed by RT-PCR and Sanger sequencing of PBMC-derived RNA of the proband. The Sanger sequencing revealed a normal splicing isoform for the wild-type allele and abnormal splicing for the mutant allele (Figure 5(a)). We detected deletion of exon 14 in the *JAK3* cDNA with the c.1914G>T variant (Figure 5(a)). However, the concentration of the mutant allele appeared to be about one-third of that of normal allele (Figure 5(a)). Agarose gel electrophoresis of RT-PCR products showed two single bands, and the Sanger sequence from the gel extraction purification shows the wild type (expected 498 bp) and mutant type (expected 370 bp) with the deletion of exon 14 (128 bp) in the *JAK3* cDNA from the c.1914G>T variant (Figure 5(b)), predicting to result in a truncated protein (p.Leu596Arg fs Ter22) (Figure 5(c)).

### 3.4. Splicing Study of *JAK3* c.1914G>T by Minigene Assay

We also conducted a minigene analysis of the wild and mutant types carrying *JAK3* c.1914G>T to further characterize the abnormal splicing. Agarose gel electrophoresis of RT-PCR products showed a single band from the wild type (expected 546 bp) and mutant type (expected 418 bp) (Figure 2(a)). The Sanger sequencing showed a normal splicing isoform for the wild type and abnormal splicing with exon 14 deletion (128 bp) for the mutant type (Figure 2(b)) which was in accordance with the splicing study from proband blood. Our functional data therefore disagreed with the prediction by the online RNA Splicer tool and SpliceAI (Figure 2(c)).

### 3.5. Analyzing the Variant Pathogenicity according to ACMG

The c.1048C>T (p.R350W) variant has been previously reported in two SCID patients (PS1) [6]. The frequency of the variant in the affected population (two SCID patients with the variant) is significantly higher than that in the control population (in-house database, PS4), and the frequency for the variant in the population database is zero (PM2). Multiple bioinformatic analyses predicted that the variant had deleterious effects, including conservation prediction, SIFT, PolyPhen_2, MutationTaster, and GERP+ (PP3). Additionally, the pathogenic variants are in trans consistent with the genetic laws of recessive genetic disease (PM3). According to the ACMG guidelines, the c.1048C>T (p.R350W) variant (PS1+PS4+PM2+PM3+PP3) was classified as a pathogenic variant.

In our study, we show that the variant c.1914G>T (p.L638=) could result in a truncated protein (p.Leu596Arg fs Ter22) (PVS1). The frequency for the novel variant in the population database is zero (PM2), and the variant from the father is conforming to the genetic laws of recessive genetic diseases (PM3). According to the ACMG guidelines, the c.1914G>T (p.L638=) variant (PVS1+PM2+PM3) was also classified as pathogenic variant.

Combining the clinical features and genetic analysis results, the infant was finally diagnosed as severe combined immunodeficiency (SCID) with T-cell-negative, B-cell-positive, and NK-cell-negative findings caused by JAK3 function defection.

## 4. Discussion

SCID is classified as a primary immunodeficiency characterized by impaired T lymphocyte differentiation. The definitive therapies of most SCID are based on gene therapy of hematopoietic stem cell transplantation (HSCT) with a 90% successful chance [7]. However, there are some serious adverse effects, such as graft rejection, graft versus host disease (GVHD), chronic infection, thymus problems, or lifelong immunoglobulin substitution [8, 9]. In recent years, the invention of induced pluripotent stem cell (iPSC) technology-based gene therapy meeting severe combined immunodeficiency has allowed scientists to cure the disorder by creating a healthy cell line from the patients in ex vivo conditions [9, 10]. Supportive care, such as antimicrobial agents and intravenous immunoglobulin (IVIG), is required for SCID to support life in the course of critical illness. However, our patient just received supportive treatment and did not pass the critical period of the disease and died, so timely diagnosis and treatment of this disease is crucial.

By Jan 2023, there were 86 variants reported being related to immunodeficiency in HGMD database (138 variants in professional HGMD database). We have added the location of the point variants in Figure 1. Most of missense mutations (30/36) were scattering in the FERM and JH2 domains, and all of the truncating mutations (20) caused by the point variants were scattered across four domains, FERM, SH2, JH2, and JH1. In our study, the variant c.1048C>T (p.R350W) reported in one SCID patient [6] was located in the FERM domain. Otherwise, the variant c.1914G>T (p.L638=) which could result in the deletion of exon 14 (128 bp) in the *JAK3* cDNA predicted to result in a truncated protein (p.Leu596Arg fs Ter22) was located in the JH2 domain (Figure 1).

In our clinical work, we have found one infant suffering CMV infection and sustained fever with little T lymphocyte and abnormal B lymphocyte function. Through WES, we found the infant carriers' two variants, a missense mutation c.1048C>T (p.R350W) inherited from her mother and the novel synonymous variant c.1914G>T (p.L638=) inherited from her father. Although the missense mutation c.1048C>T (p.R350W) was previously reported in a SCID patient [6], the pathogenicity was not verified. In our study, we predicted that the amino acid p.R350 is highly conservative in multiple species, and the structural analysis illustrates that acidic amino acid tryptophan substitution could alter the side strand structure in which the original arginine is alkaline amino acid. Otherwise, p.R350 was sited at JH4 domain, and the p.W350 variant may lose the regulatory catalytic function, and the c.1048C>T (p.R350W) variant was predicted to be deleterious by bioinformatic analysis. Whether the missense variant could impair the catalytic function in vivo or in vitro needs to be further studied.

Synonymous mutations are frequently detected from gene sequencing and considered to be nonpathogenic as they do not alter the amino acid of the encoded protein. However, synonymous mutations could also lead to abnormal splicing by creating or altering noncanonical splicing sites, and there are quite a few reports about synonymous variations contributing to abnormal splicing [11]. In our study, the proband has the synonymous variant c.1914G>T (p. L638=) in the *JAK3* gene which is inherited from her mother, with location at the 5′ end of exon 14, in which the nucleoside site is against the splicing donor site of the 5′ end of the intron which starts with the G. We speculated that the variant could disturb the donor splice site function. The mRNA detection of peripheral blood samples from the patient and minigene assay of the synonymous variant showed the deletion of exon 14 in the *JAK3* cDNA from the c.1914G>T variant. The cDNA level with the c.1914G>T mutation results in only up to about 1/3 of the normal cDNA strand. This is similar to our previous synonymous mutation research [11], about which the minigene assay confirmed the nonsense-mediated mRNA decay (NMD), mediated downregulation, which contributes to about one-third of disease-causing mRNAs [12, 13]. However, no possible abnormal splice patterns were predicted through splicing prediction software.

The molecular diagnosis for SCID patients is pivotal not only for the proband but also for the family, as to provide precise genetic counseling and execute the prenatal diagnosis to deliver a healthy child. In addition, preemptive stem cell transplantation and intrauterine transplantation with hematopoietic stem cell could deliver a healthy baby for the SCID family with the *JAK3* variant.

## Data Availability

The data that support the findings of this study are available on request from the corresponding authors. The data are not publicly available due to privacy or ethical restrictions.

## Supplementary Materials

Supplementary 1 Supplementary Figure 1: JAK3 is highly expressed on peripheral whole blood referenced by UCSC.

Supplementary 2 Supplementary Figure 2: pathogenicity prediction of the synonymous mutation c.1914G>T (p.L638=) of JAK3. The synonymous mutation c.1914G>T (p.L638=) of JAK3 is nonpathogenic predicted by the online RNA Splicer tool.
