# Supplementary figures and images for: Combination of Synonymous and Missense Mutations in JAK3 Gene Contributes to Severe Combined Immunodeficiency in One Child
Source: Hum Mutat. 2023 Sep 13;2023:6633251. doi: 10.1155/2023/6633251 (PMC11919225; doi:10.1155/2023/6633251)

ENSG00000105639.14 Gene Expression from GTEx (Release V6)

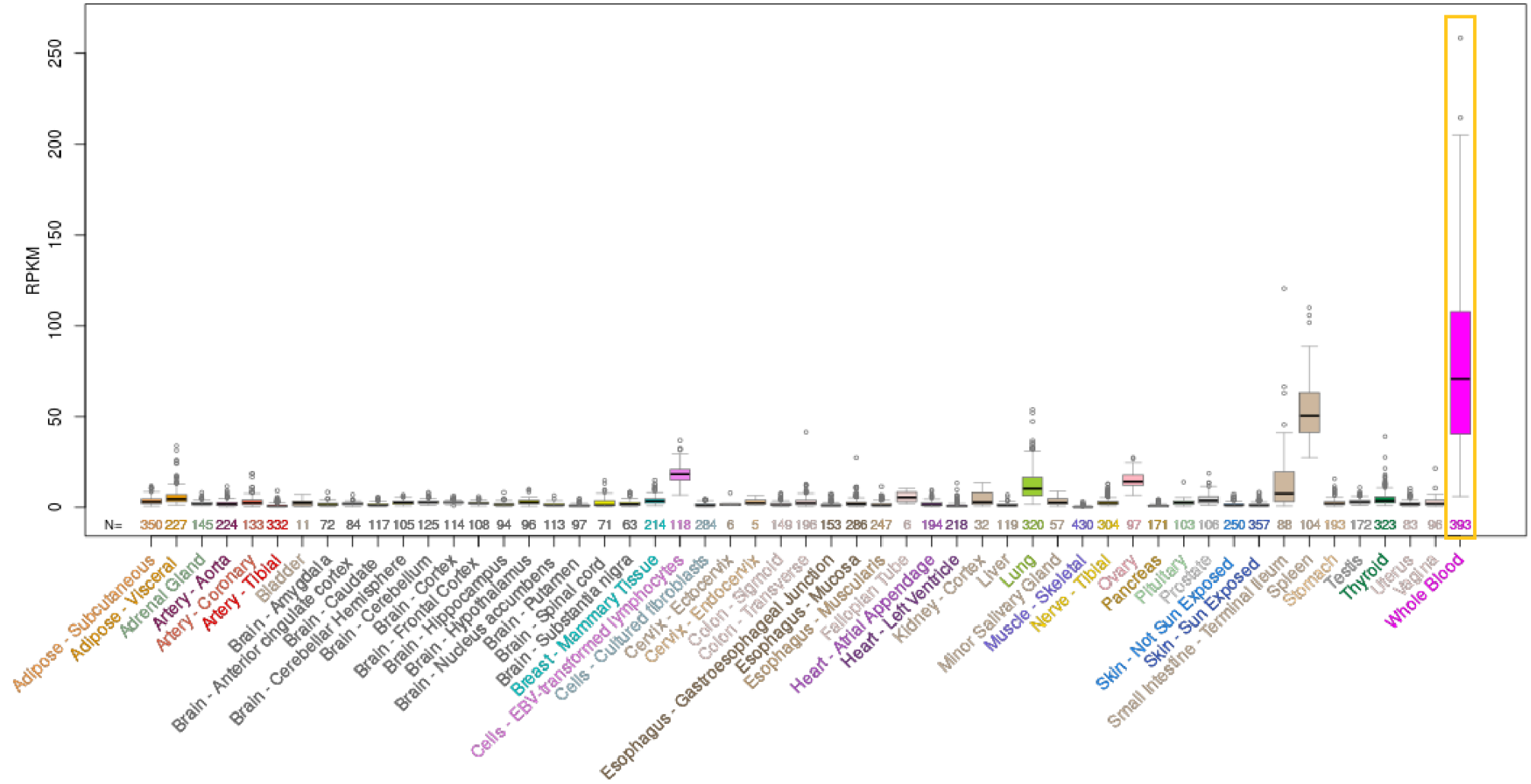

Supplement: Supplementary 1 — Supplementary Figure 1: JAK3 is highly expressed on peripheral whole blood referenced by UCSC. [file 6633251.f1.pdf]
