# Supplementary material for: Combination of Synonymous and Missense Mutations in JAK3 Gene Contributes to Severe Combined Immunodeficiency in One Child
Source: Hum Mutat. 2023 Sep 13;2023:6633251. doi: 10.1155/2023/6633251 (PMC11919225; doi:10.1155/2023/6633251)

Prediction Result

Check orthologous mutation | Mus musculus | Rattus norvegicus

human JAK3(NM_000215.4, c.1914G>T)

Splice Pattern 1: Consistent with the wild-type RNA splicing pattern

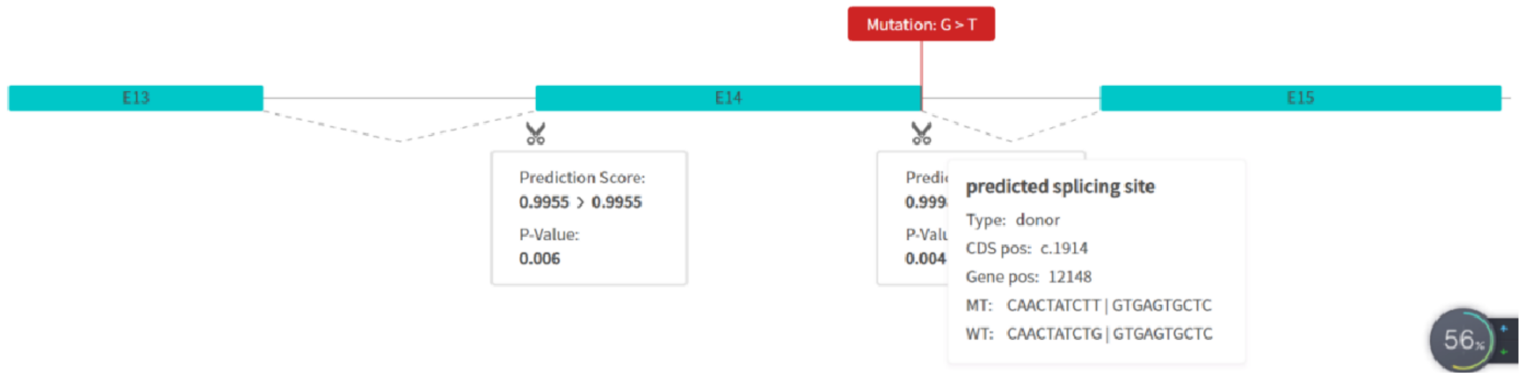

Supplement: Supplementary 2 — Supplementary Figure 2: pathogenicity prediction of the synonymous mutation c.1914G>T (p.L638=) of JAK3. The synonymous mutation c.1914G>T (p.L638=) of JAK3 is nonpathogenic predicted by the online RNA Splicer tool. [file 6633251.f2.pdf]
